# Molecular Mobility of *N*‐Acetylgalactosamine‐Modified Cyclodextrins on a Polyrotaxane for Highly Efficient Liver Targeting of Antibody Chimeras and Genome‐Editing Ribonucleoproteins

**DOI:** 10.1002/advs.75996

**Published:** 2026-06-11

**Authors:** Toru Taharabaru, Keiichi Motoyama, Yuting Wen, Zhongxing Zhang, Xuehao Tian, Jun Li, Taishi Higashi

**Affiliations:** ^1^ Graduate School of Pharmaceutical Sciences Kumamoto University Kumamoto Japan; ^2^ Department of Biomedical Engineering College of Design and Engineering National University of Singapore Singapore Singapore

**Keywords:** antibody chimeras, genome‐editing ribonucleoproteins, molecular mobilities, polyrotaxanes, *N*‐Acetylgalactosamine

## Abstract

Triantennary *N*‐acetylgalactosamine (*tri*GalNAc), which interacts strongly with the trimeric structure of asialoglycoprotein receptors (ASGPRs), is a validated platform for liver targeting. However, the intricate design and synthesis of its linkers impose high production costs and significant technical challenges. In this study, we report an alternative strategy for targeting ASGPR using monovalent GalNAc (*mono*GalNAc) conjugated to the cyclic molecules of polyrotaxane, which can rotate and translocate along the axial polymer chain. The intracellular uptake efficacy of *mono*GalNAc‐modified polyrotaxane is comparable to that of *tri*GalNAc‐modified polyrotaxane and significantly higher than that of *tri*GalNAc‐ or *mono*GalNAc‐modified immobile control polymers. These results suggest that the inherent mobility of polyrotaxanes allows *mono*GalNAc moieties to cluster in a trivalent‐like manner, thereby enhancing multivalent interactions with multiple ASGPR oligomers. The successful application of *mono*GalNAc‐modified polyrotaxane to lysosome‐targeting antibody chimeras and genome‐editing nanoparticles demonstrates that this facile technology is a highly promising alternative to conventional *tri*GalNAc.

## Graphical abstract

Monovalent *N*‐acetylgalactosamine (GalNAc)‐modified polyrotaxane enables efficient liver targeting by utilizing ligand mobility. The sliding and rotating cyclic components i.e., cyclodextrin in the polyrotaxane dynamically cluster GalNAc moieties, thereby mimicking trivalent interactions with asialoglycoprotein receptors. This system outperforms conventional triantennary GalNAc ligands and simplifies molecular design, offering a versatile platform for delivering antibody chimeras and genome‐editing ribonucleoproteins.

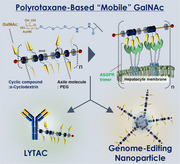

## 1 Introduction

*N*‐Acetylgalactosamine (GalNAc), which is the ligand for liver asialoglycoprotein receptors (ASGPR), is the most extensively utilized active targeting ligand in contemporary biomedical research [1, 2, 3]. Approximately 500 000 copies of ASGPRs express in hepatocytes [4, 5, 6, 7], and 5%–10% of them express on the surface of hepatocytes [5, 8]. When ASGPRs interact with their ligands, they get rapidly internalized into the cells and recycled to the cell surface in 5–10 min [9, 10]. The interaction between GalNAc and ASGPRs is ∼50 times higher than that between galactose and ASGPR [11, 12]; therefore, GalNAcs are utilized as active targeting ligands for drug delivery systems. The ASGPR is a hetero oligomer receptor of ASGR1 and ASGR2 with molar ratio 2:1 [13, 14, 15]. When the GalNAc‐ligand configuration is clustered within 10–25 Å, the interaction between ASGPRs and trivalent GalNAcs is maximized (4 = 3 > 2 > 1 GalNAc(s)) [11, 12, 16, 17]. Thus, the trimer model of ASGPRs has been proposed [18], and the triantennary GalNAc (*tri*GalNAc), which is aligned with the structural conformation of the ASGPR trimer, has been developed [19]. To date, several drugs containing *tri*GalNAc directly conjugated to small interfering RNAs (siRNAs) or antisense oligonucleotides (ASOs) have received regulatory approval, firmly establishing *tri*GalNAc as a key platform for liver‐targeted drug delivery [1, 2, 3].

However, the intricate nature of the linker design and synthesis of *tri*GalNAc imposes significant technical challenges. Moreover, *tri*GalNAc and *tri*GalNAc‐based drug delivery systems present several limitations that must be addressed. *Tri*GalNAc is suitable for nucleic acid drugs such as siRNA and ASO, with a molecular weight (M.W.) of up to ∼15 kDa, however, the delivery capacity of *tri*GalNAc for macromolecules tends to be modest. In fact, the intracellular uptake of *tri*GalNAc‐siRNA is more than 10 times greater than that of unmodified siRNA [19], whereas in the case of genome‐editing molecules (Cas9 ribonucleoprotein: Cas9 RNP, ∼190 kDa), the enhancement effect is only around half that of siRNA, despite improvement in the interaction with ASGPRs through the derivatization of *tri*GalNAc to artificial sugar [20, 21]. Furthermore, in the *tri*GalNAc‐antibody conjugate, the Fab fragment conjugate offers greater advantage in terms of intracellular delivery compared with the full‐length antibody conjugate [22].

Importantly, emerging evidence indicates that conventional *tri*GalNAc ligands can be improved by reconsidering their design strategies. For example, Schmidt et al. reported that GalNAcs aligned at 60 Å in length, which can interact with multiple ASGPR oligomers, show the superior efficacy to *tri*GalNAc or clustered GalNAcs [23]. Additionally, Yamamoto et al. showed that monovalent GalNAc (*mono*GalNAc) modified in an oligonucleotide unit can enhance the efficacy in a modification number dependent manner at least in the range from monomer to hexamer [24]. These findings suggest the contribution of the multivalent interaction with multiple ASGPR oligomers and/or minor oligomers such as hexamers [15] other than the trimer of the ASGPR.

A polyrotaxane (PRX) is a mechanically interlocked supramolecular polymer consisting of a linear axile molecule threaded multiple cyclic molecules with sterically hindered by bulky endcaps. α‐Cyclodextrin (αCD) and polyethylene glycol (PEG) are often use as a cyclic molecule and an axile molecule, respectively [25, 26, 27]. CD‐based PRXs have a number of advantages, such as high yield by facile preparation, favourable safety profile, and low cost. Therefore, CD‐based PRXs have been broadly applied as biomaterials [28, 29, 30, 31]. Most importantly, CD‐based PRXs are highly flexible and exhibit mobile properties, i.e. CDs in the PRX can rotate and translocate along the axile [32]. Utilizing this mobility, CD‐functionalized PRXs have been developed, such as aminated PRXs [33, 34, 35, 36, 37] and ligand‐modified PRXs [38, 39, 40, 41]. Ligand‐modified PRXs enhance the multivalent interaction of modified ligands and target receptors by avoiding spatial mismatches during interaction. Ohashi et al. developed CD‐based PRXs modified with clustered GalNAc, which were modified with three or more GalNAcs per CD, and reported that the interaction between ASGR1 and PRXs increased in a modification ratio of GalNAcs‐dependent manner [41]. However, this report describes an example of multiple GalNAcs grafted onto the CDs, and the effect of the mobility of *mono*GalNAcs in the PRX in terms of their interaction mode with the ASGPR trimer/oligomer has not been investigated or demonstrated. In other words, from the perspective of developing simpler and more efficient GalNAc ligands, neither application studies on PRXs nor strategies have been reported for enhancing interactions with the trimeric or oligomeric structures of ASGPRs by utilizing the inherent molecular mobility of PRXs.

**FIGURE 1:**
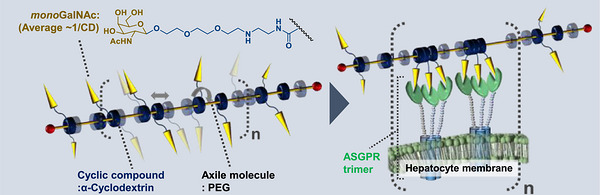
Schematic illustration of *mono*GalNAc‐PRX and the interaction mode of *mono*GalNAc‐PRX and ASGPRs.

In this study, we hypothesize that modifying the CDs in the PRX with *mono*GalNAc allows recognition and response to the oligomer structure and the distribution of multiple ASGPR oligomers on the surface of hepatocytes, thereby inducing modified *mono*GalNAc clustering in a trivalent‐like manner and efficient multivalent interactions (Figure 1). This approach can simplify the structural design and synthesis of GalNAc ligands and is expected to serve as a next‐generation structural design that enables multivalent interactions with multiple ASGPR oligomers and accommodates minor oligomers of the ASGPR. To validate these hypotheses, we synthesized *mono*GalNAc‐modified PRX (*mono*GalNAc‐PRX), *tri*GalNAc‐modified PRX (*tri*GalNAc‐PRX), and the corresponding immobile control polymers and conducted comparative studies of the synthesized polymers. Furthermore, we also hypothesize that the development of GalNAc‐PRX‐based drug delivery systems can help to facilitate improvements to the efficacy of macromolecule delivery, which is insufficient when using the conventional *tri*GalNAc system. Therefore, drug conjugates and nanoparticles functionalized with GalNAc‐PRXs were developed to achieve a promising liver‐targeting drug delivery system for Cas9 RNP and antibody chimeras.

## 2 Results and Discussion

### 2.1 Synthesis and Cellular Internalization of GalNAc‐Modified Polymers

From a biological perspective, αCD exhibits lower hemolytic activity compared to βCD, offering superior safety and biocompatibility as a drug delivery vehicle. Furthermore, αCD‐based PRX can be synthesized high yield through a facile preparation method. Therefore, in this study, αCD‐based PRXs were employed due to their distinct advantages in safety and synthetic efficiency.

**FIGURE 2:**
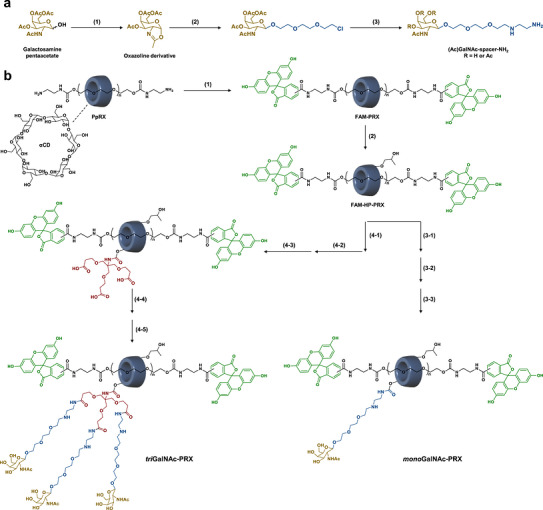
Preparation pathway of GalNAc‐PRXs. (a) GalNAc‐spacer‐NH_2_. (1) trimethylsilyl trifluoromethanesulfonate (TMSOTf), in dry dichloromethane (DCM), 18 h, 50°C; (2) 2‐[2‐(2‐chloroethoxy)ethoxy]ethanol, TMSOTf, in in dry DCM, 18 h, r.t.; (3) Excess ethylenediamine, in dry DMSO, 18 h, 50°C. (b) Fluorescent‐labeled GalNAc‐PRXs. (1) FAM‐COOH, benzotriazol‐1‐yloxytris(dimethylamino)phosphonium hexafluorophosphate (BOP) reagent, *N*‐ethyldiisopropylamine (EDIPA), in dimethylformamide (DMF), 18 h, on ice; (2) Propylene oxide, in 1N NaOHaq, overnight, on ice; (3‐1) CDI, in dry dimethyl sulfoxide (DMSO), overnight, 25°C; (3‐2) (Ac)GalNAc‐spacer‐NH_2_, in dry DMSO, 6 h, 25°C; (3‐3) in 1N NaOHaq for *O*‐deacetylation, 2 h, on ice; (4‐1) CDI, in dry DMSO, overnight, 25°C, (4‐2) Tris[[2‐(tert‐butoxycarbonyl)ethoxy]methyl]methylamine, in dry DMSO, overnight, 25°C, (4‐3) Trifluoracetic acid for deprotection, in DMSO, 1 h, r.t.; (4‐4) (Ac)GalNAc‐spacer‐NH_2_, *N*‐hydroxysuccinimide, 1‐(3‐Dimethylaminopropyl)‐3‐ethylcarbodiimide hydrochloride, Triethylamine, in dry DMSO, 9 h, 25°C; (4‐5) in 1N NaOHaq for *O*‐deacetylation, 2 h, on ice.

**FIGURE 3:**
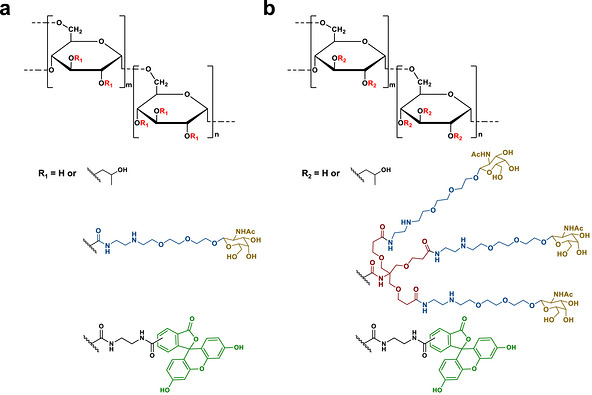
Chemical structures of (a) *mono*GalNAc‐DEX and (b) *tri*GalNAc‐DEX.

To compare the internalization to hepatocytes and interaction with ASGPRs, we first synthesized *mono*GalNAc‐PRX and *tri*GalNAc‐PRX as well as immobile control polymers (dextran: DEX) (Figures 2, 3, and Figures S1–S15). GalNAc‐spacer‐NH_2_ was synthesized using galactosamine pentaacetate as the starting material based on the scheme shown in Figure 2a. In order to suppress non‐specific adsorption by modifying numerous ligands to the polymer, short oligoethylene glycol chains were used as spacers between a GalNAc and the polymers instead of the conventional oligoalkyl chain [19] with a similar molecular length. To detect cellular internalization based on fluorescence intensity, 5(6)‐Carboxylfluorescein (FAM‐COOH) was used as the endcap of the PRX (Figure 2b). The formation of the mechanically interlocked structure was confirmed by 2D‐NMR (NOESY), where clear cross‐peaks between PEG and the anomeric proton of αCDs were observed (Figures S7 and S10). FAM‐COOH was modified to DEXs with an ethylenediamine linker to obtain the equal fluorescence intensity to that of PRXs (Figure 3 and Figure S12). *Mono*GalNAc‐PRX and *mono*GalNAc‐DEX were prepared by the direct conjugation of GalNAc‐spacer‐NH_2_ to the hydroxyl group of CD or DEX via *N,N*‐Carbonyldiimidazole (CDI). To prepare *tri*GalNAc‐PRX or *tri*GalNAc‐DEX, GalNAc‐spacer‐NH_2_ was modified via a forked linker used for commercial *tri*GalNAc [19]. The average number of modified GalNAc to each polymer was determined via the integral value of the anomeric proton of GalNAcs to that of CDs or DEX in ^1^H NMR spectra as well as the number of FAM to each polymer (Figures S9, S11, S14, and S15). Although CDs contain multiple hydroxyl groups, the modification ratio of GalNAc/CD could be adjusted by varying the molar ratios of CDI and (Ac)GalNAc‐spacer‐NH_2_ to the CD unit (Table S1), and the modification ratio of *mono*GalNAc/CD was adjusted to ∼1. In the case of *tri*GalNAc‐PRX, one *tri*GalNAc unit was modified per three CD. Therefore, the total number of GalNAc units per PRXs molecule was comparable between *mono*GalNAc‐PRX and *tri*GalNAc‐PRX. Furthermore, the GalNAc‐modified polymers in this study were confirmed to have similar numbers of modified GalNAcs and M.W.s (Table 1) as well as fluorescence intensities. In particular, *mono*GalNAc‐PRX offers significant advantages in terms of synthesis, as its synthesis process is significantly reduced and simplified compared with that of the conventional solid‐supported *tri*GalNAc (Figure S16) [19].

**TABLE 1:** GalNAc‐modified polymers were characterized via ^1^H NMR. The number of αCD in the PRXs were calculated based on the integral value of the anomeric proton of αCD and ethylene glycol proton of PEG. The modification ratio of GalNAcs was calculated based on the integral value of the anomeric proton of αCD or DEX and the anomeric proton of GalNAc. M.W. of each polymer was calculated based on the chemical composition determined by ^1^H NMR. The number of αCD, coverage (%), and GalNAc/αCD or polymer of modification ratio of GalNAcs in this table are average value.

Polymer	No. of αCD	Coverage (%)	*mono*GalNAc/αCD	*mono*GalNAc/polymer	αCD/*tri*GalNAc	*tri*GalNAc/polymer	M.W. (kDa)
*mono*GalNAc‐PRX	49	21.6	0.9	44	—	—	100
*mono*GalNAc‐DEX	—	—	—	44	—	—	110
*tri*GalNAc‐PRX	49	21.6	—	—	3.3	15	103
*tri*GalNAc‐DEX	—	—	—	—	—	15	107

**FIGURE 4:**
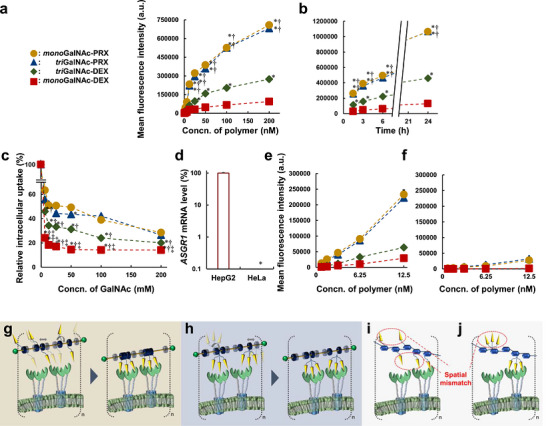
Intracellular uptake of GalNAc‐modified polymers. (a) Intracellular uptake in HepG2 cells at various concentration after 3 h of treatment and (b) various time courses of 50 nm treatment. n = 3, *p < 0.001 *vs*. *mono*GalNAc‐DEX, †*p* < 0.001 vs. *tri*GalNAc‐DEX. (c) Relative intracellular uptake efficacies of GalNAc‐modified polymers in HepG2 cells in the presence of free GalNAc. Intracellular uptake efficacy of each polymer without free GalNAc was set at 100%. [Polymer] = 25 nm. n = 3, *p < 0.001 *vs*. *mono*GalNAc‐PRX, †*p* < 0.001 *vs*. *tri*GalNAc‐PRX, ‡*p* < 0.001 *vs*. *tri*GalNAc‐DEX. (d) *ASGR1* mRNA expression levels of HepG2 and HeLa cells. Relative expression level of human *ASGR1*/*GAPDH* mRNA of HepG2 cells was set at 100%. n = 3, *p < 0.001 *vs*. HepG2 cells. (e, f) Comparison of intracellular uptake of GalNAc‐modified polymers in **e**, HepG2 cells (expansion view of (a) at 0–12.5 nm) and (f) HeLa cells after 3 h of treatment (n = 3). (g, h, i, j) Proposed mechanism of interaction between ASGPRs and modified GalNAcs in (g) *mono*GalNAc‐PRX, (h) *tri*GalNAc‐PRX, (i), *mono*GalNAc‐DEX, and (j) *tri*GalNAc‐PRX.

The intracellular uptake of GalNAc‐modified polymers was assayed in ASGPR‐positive hepatocytes, a human liver cancer cell line (HepG2 cells) (Figure 4). The cellular internalization of the GalNAc‐modified polymers increased in a concentration‐ and treatment time‐dependent manner (Figure 4a,b). The intracellular‐uptake efficacy of *tri*GalNAc‐DEX in HepG2 cells was significantly greater than that of *mono*GalNAc‐DEX under all experimental conditions in this study. In contrast, *mono*GalNAc‐PRX and *tri*GalNAc‐PRX exhibited comparable intracellular‐uptake efficiencies in HepG2 cells. Most importantly, the intracellular‐uptake efficacies of *mono*GalNAc‐PRX and *tri*GalNAc‐PRX were significantly higher than those of *tri*GalNAc‐DEX and *mono*GalNAc‐DEX.

Meanwhile, the intracellular uptake of the GalNAc‐modified polymers in HepG2 cells was decreased by the addition of free GalNAc (Figure 4c). Moreover, the GalNAc‐modified polymers showed negligible uptake in HeLa cells, which are ASGPR‐negative cells (Figure 4d–f). The results suggested that GalNAc‐modified polymers were internalized to HepG2 cells via the ASGPR. Additionally, the competitive inhibition study suggested that the strength order of the interaction between the polymers and ASGPR was the same as that of the intracellular‐uptake efficacies (Figure 4c).

The finding that *tri*GalNAc‐DEX demonstrated a higher intracellular‐uptake efficacy than *mono*GalNAc‐DEX supports the rationality of the conventional *tri*GalNAc design to some extent. Importantly, both *mono*GalNAc‐PRX and *tri*GalNAc‐PRX showed significantly higher efficacies than *tri*GalNAc‐DEX, thereby suggesting that the molecular mobility of CDs on PRX adjusts the presentation of modified GalNAcs to ASGPRs without any spatial mismatches (Figure 4g–j). Additionally, hence *mono*GalNAc‐PRX and *tri*GalNAc‐PRX exhibited comparable efficacies, the modified *mono*GalNAcs in the CDs on PRX clustered in a trivalent‐like manner (Figure 4g,h). These findings support our hypotheses and suggest that the molecular mobility of CDs on PRX enables highly efficient and facile multivalent interactions between modified GalNAcs and ASGPRs. The stoichiometry of ASGR1 and ASGR2 is commonly reported to be 2:1; however, some studies have indicated a broader range of 2:1 to 5:1 [13, 14, 15]. These findings suggest that ASGPRs may exist as oligomeric aggregates of two to five ASGR1 and one ASGR2 loosely associated subunits [23], and the oligomers other than the canonical trimer would be involved in the cellular uptake of *mono*GalNAc‐PRX. The cavity height of CD is 7.9 Å, which is suitable for clustering GalNAcs to both highly clustered (∼25 Å) structures like a trivalent structure and loosely associated tetra to hexamer‐like structures. A comprehensive understanding of this interaction mechanism is crucial for developing more potent GalNAc‐PRX‐based ligands. Recently, a chemical strategy for modifying GalNAc into artificial sugar was reported to enhance interaction with ASGPRs [21]. Combining such chemical approaches with the mobile properties of PRX can materialize the development of more robust and highly potent GalNAc ligands.

Based on these results, in the following study, feasibility of the *mono*GalNAc‐PRX‐based drug delivery system was explored.

### 2.2 *Mono*GalNAc‐PRX‐Based Lysosome‐Targeting Antibody Chimeras (LYTACs)

LYTACs are a new class of bifunctional molecules designed for the degradation of targeted proteins [42, 43]. A typical LYTAC molecule comprises an antibody that captures the targeted protein (antigen) and another ligand that binds to a cell‐surface receptor. LYTACs capture target antigens and are internalized by target cells via endocytosis, as initiated by modified ligands. Consequently, this complex is trafficked to the lysosomes, where the antigen is degraded by lysosomal functions. The strategic use of ASGPRs for targeted protein degradation by LYTACs is highly advantageous. ASGPRs originally constituted the catabolic machinery and direct the process to the liver, which is the largest organ in the body and a highly efficient center for a vast number of metabolic and degradative functions. Therefore, LYTACs consisting of *tri*GalNAc and antibodies were developed for the efficient targeting of liver hepatocytes via ASGPRs [22, 44, 45]. In this study, GalNAc‐PRX/LYTAC conjugate (GalNAc‐PRX‐LYTAC) was synthesized and evaluated in order to investigate the potential of GalNAc‐PRX‐based LYTAC as a highly promising alternative to *tri*GalNAc‐based LYTACs.

**FIGURE 5:**
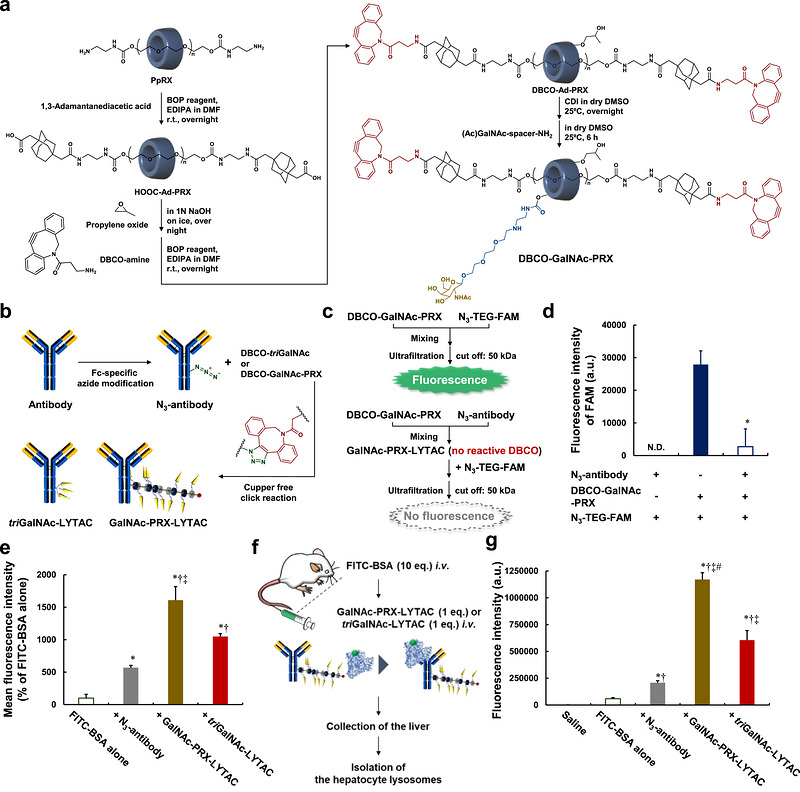
Development of GalNAc‐PRX‐LYTAC. (a) Preparation pathway of DBCO‐GalNAc‐PRX. (b) Preparation pathway and schematic illustration of GalNAc‐PRX‐LYTAC and *tri*GalNAc‐LYTAC. (c, d) Reactivity of DBCO‐GalNAc‐PRX with N_3_‐TEG‐FAM and N_3_‐antibody. n = 3, *p < 0.05 *vs*. N_3_‐antibody (‐) DBCO‐GalNAc‐PRX (+) N_3_‐TEG‐FAM (+). (e) Intracellular uptake of FITC‐BSA with N_3_‐antibody, GalNAc‐PRX‐LYTAC, or *tri*GalNAc‐LYTAC. [FITC‐BSA] = 100 nm. [Antibody] = 10 nm. n = 6, *p < 0.05 *vs*. FITC‐BSA alone, †*p* < 0.05 *vs*. + N_3_‐antibody, ‡*p* < 0.05 *vs*. + *tri*GalNAc‐LYTAC. (f, g) In vivo activities of LYTACs. (g) Accumulation of FITC‐BSA in hepatocyte lysosomes after intravenous administration of LYTACs to mice. n = 6, *p < 0.05 *vs*. saline, †*p* < 0.05 *vs*. FITC‐BSA alone, ‡*p* < 0.05 *vs*. + N_3_‐antibody, #*p* < 0.05 *vs*. + *tri*GalNAc‐LYTAC.

As shown in Figure 5a, to prepare dibenzocyclooctyne (DBCO)‐end *mono*GalNAc‐PRX (DBCO‐GalNAc‐PRX) for the cupper‐free click reaction with the azide (N_3_) moiety of the antibody, acetic acid‐terminated adamantane (Ad)‐end PRX (HOOC‐Ad‐PRX) was synthesized (Figures S17 and S18). Subsequently, the carboxylic acids at the PRX terminals were modified with DBCO‐amine (Figure S19). The hydroxyl groups of CDs in the resulting DBCO‐Ad‐PRX were directly conjugated with GalNAc‐spacer‐NH_2_. The obtained DBCO‐GalNAc‐PRX was confirmed that ∼1 *mono*GalNAc was modified in each CD (Figures S20, S21 and Table S2). For the Fc‐specific N_3_‐modification of the antibody, the carbohydrate groups of the antibody were enzymatically modified with N_3_‐sugar (Figure S22). GalNAc‐PRX‐LYTAC was prepared by mixing DBCO‐GalNAc‐PRX and the N_3_‐antibody in phosphate‐buffered saline as well as conventional *tri*GalNAc‐LYTAC (Figure 5b, Figure S23).

The reactivity of DBCO in DBCO‐GalNAc‐PRX and the reaction of DBCO‐GalNAc‐PRX and the N_3_‐antibody were confirmed using an N_3_‐triethylene glycol (TEG)‐FAM‐based assay (Figure 5c,d). The fluorescence derived from the FAM of the mixture of DBCO‐GalNAc‐PRX and N_3_‐TEG‐FAM after ultrafiltration (M.W. cut off: 50 kDa) was observed, indicating the reactivity of DBCO‐GalNAc‐PRX with the N_3_‐compound. Furthermore, the fluorescence was significantly decreased by the addition of the N_3_‐antibody at a molar ratio of DBCO‐GalNAc‐PRX: N_3_‐antibody = 1:1 before incubation with N_3_‐TEG‐FAM. This result is further supported by ^1^H NMR integration data (Figures S19 and S20), which suggested the complete conjugation of DBCO and the preservation of its reactivity following the GalNAc modification. The results also suggest that almost all the DBCO in DBCO‐GalNAc‐PRX reacted with the N_3_‐antibody under these conditions. Furthermore, GalNAc‐PRX‐LYTAC showed slightly increased particle size compared to N_3_‐antibody and DBCO‐GalNAc‐LYTAC, and no aggregates were detected (Figure S24). These results suggest that GalNAc‐PRX‐LYTAC was successfully prepared.

To evaluate the in vitro activity of GalNAc‐PRX‐LYTAC, HepG2 cells were treated with GalNAc‐PRX‐LYTAC (anti‐bovine serum albumin: BSA) and FITC‐labeled BSA (FITC‐BSA) (Figure 5e). The results showed that GalNAc‐PRX‐LYTAC delivered the antigen, namely FITC‐BSA with high efficacy. Most importantly, the efficacy of GalNAc‐PRX‐LYTAC was significantly greater than that of conventional *tri*GalNAc‐LYTAC. The antigen delivering efficacy of GalNAc‐PRX‐LYTAC was decreased in the presence of free GalNAc (Figure S25). Consistent with this result, GalNAc‐unmodified‐PRX LYTAC markedly lower LYTAC efficacy than GalNAc‐PRX‐LYTAC (Figure S25). Additionally, the intracellular uptake of FAM‐labeled DBCO‐GalNAc‐PRX was significantly higher than that of FAM‐labeled *tri*GalNAc (Figure S26). These results suggest that highly efficient ASGPR‐mediated intracellular uptake of GalNAc‐PRX, the targeting module of LYTAC, contributes to the efficient antigen delivery of GalNAc‐PRX‐LYTAC.

Furthermore, FITC‐BSA was intravenously administrated to the healthy mice, and subsequently LYTACs were administrated. After the injections, the liver was collected, and the lysosomes of the hepatocytes were isolated to investigate whether GalNAc‐PRX‐LYTAC can capture the antigens and deliver them to the hepatic lysosomes in vivo (Figure 5f,g). The results showed that GalNAc‐PRX‐LYTAC successfully captured antigens in the blood and delivered them to hepatic lysosomes for digestion with significantly higher efficacy than conventional *tri*GalNAc‐LYTAC, even in vivo circumstances. These results suggest that GalNAc‐PRX‐LYTAC may have the potentials as a novel class of LYTAC for targeting the liver.

### 2.3 Functionalization of Genome‐Editing Nanoparticle With *Mono*GalNAc‐PRX

Clustered regularly interspaced short palindromic repeats (CRISPR)‐CRISPR‐associated protein 9 (Cas9) is a robust and facile genome editing system [46]. To induce genome editing via CRISPR‐Cas9 system, the introduction of enzyme for DNA cleavage (Cas9) and single‐guide RNA (sgRNA) for target recognition are necessary. Gene transfection by plasmid DNA or mRNA was applied for the introduction of these two compounds; however, the direct cytosolic delivery of the Cas9/sgRNA complex (Cas9 RNP) is the most promising approach [47, 48] and is utilized for genome‐editing‐based ex vivo cell therapies [49], which are regulatory approved. Despite these advantages, no therapeutics exist for Cas9 RNP in vivo because of delivery issues, such as cytosolic and targeted delivery. Focusing on the targeted delivery of Cas9 RNP to hepatocytes, Rouet et al. developed a conjugate [20] of triantennary GalNAc‐mimicking artificial sugar [21] and Cas9 RNP; however, the genome‐editing efficacy of this system was modest because this system lacks endosomal escape and/or the intracellular uptake was not efficient to invalidate the contribution of degradation of Cas9 RNP in endo/lysosomes.

Previously, we developed aminated PRX, the 5th generation (amino‐PRX (5G)) as an intracellular delivery carrier for Cas9 RNP [35, 36, 37] amino‐PRX (5G) can interact and form complexes with Cas9 RNP only by mixing due to the mobile properties of the amino groups modified in CDs on PRX. Furthermore, the modified amino group and backbone structure can transform in accordance with the intracellular environment, such as low pH in the endosome and reducing conditions in the cytosol. Consequently, amino‐PRX (5G) shows the multistep transforming for control the intracellular dynamics of Cas9 RNP, such as endosomal escape, release, nuclear localization, and long‐term degradation. However, amino‐PRX (5G) has no targeting ability. Therefore, in this study, amino‐PRX (5G)/Cas9 RNP complex was functionalized with *mono*GalNAc‐PRX for liver targeting, and the resulting *mono*GalNAc‐PRX/amino‐PRX (5G)/Cas9 RNP was evaluated in vitro and in vivo.

**FIGURE 6:**
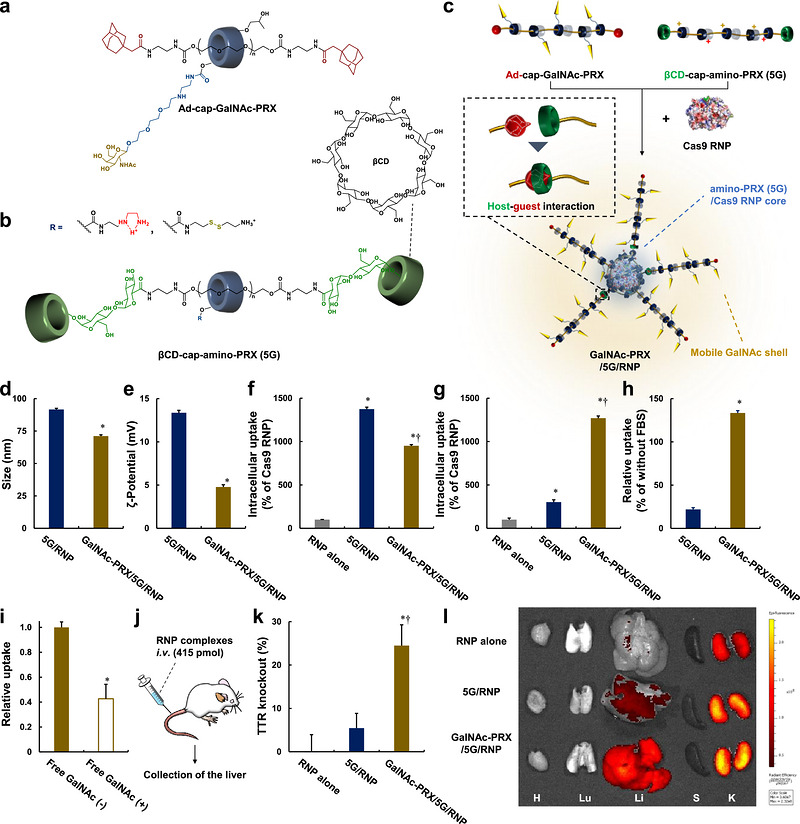
Functionalization of genome‐editing nanoparticle with GalNAc‐PRXs. (a, b) Chemical structure of (a) Ad‐cap‐GalNAc‐PRX and (b) βCD‐cap‐amino‐PRX (5G). (c) Preparation pathway and schematic illustration of GalNAc‐PRX‐functionalized Cas9 RNP complex. (d) Size and (e) ζ‐potentials of Cas9 RNP complexes. n = 3, *p < 0.05 *vs*. 5G/RNP. (f, g) Intracellular uptake of Cas9 RNP complexes (f) in the absence or (g) in the presence of FBS in HepG2 cells. [RNP] = 29.2 nm. n = 3, *p < 0.05 *vs*. RNP alone, †*p* < 0.05 *vs*. 5G/RNP. (h) Relative intracellular uptake efficacies of each Cas9 RNP complex in the presence or absence of FBS in HepG2 cells. n = 3, *p < 0.05 *vs*. 5G/RNP. (i) Effect of free GalNAc on the intracellular‐uptake efficacies of GalNAc‐PRX/5G/RNP in HepG2 cells. [RNP] = 29.2 nm. n = 3, *p < 0.05 *vs*. free GalNAc (‐). (j, k) In vivo genome‐editing efficacies of Cas9 RNP complexes after intravenous administration to mice. n = 6, *p < 0.05 *vs*. RNP alone, †*p* < 0.05 *vs*. 5G/RNP. (l) Distribution of ATTO‐550‐labeled Cas9 RNP complexes after intravenous administration to mice detected by IVIS. This figure shows representative image of three mice. H: Heart, Lu: lung, Li: liver, S: spleen, K: kidneys.

To utilize the host–guest interaction of Ad and βCD for the functionalization of amino‐PRX (5G) with *mono*GalNAc‐PRX, Ad‐capped *mono*GalNAc‐PRX (Ad‐cap‐GalNAc‐PRX) and βCD‐cap‐amino‐PRX (5G) were synthesized (Figure 6a,b, Figures S27–S32, and Tables S3, S4). The M.W. of a used axile PEG was 35 kDa, and the degree of substitution of the amino groups of amino‐PRX (5G) was adjusted to ∼1.0, as we previously optimized [37].

The Ad‐GalNAc‐PRX/βCD‐cap‐amino‐PRX (5G)/Cas9 RNP (GalNAc‐PRX/5G/RNP) was prepared by initially mixing the solution of Ad‐GalNAc‐PRX and solid βCD‐cap‐amino‐PRX (5G) at a molar ratio of 2:1, followed by a second mixing of Cas9 RNP and the polymer solution (Figure 6c). The functionalization of 5G/RNP with GalNAc‐PRX was confirmed by dynamic light scattering. As a result, reduction in the size and ζ‐potentials of 5G/RNP by GalNAc‐PRX functionalization was observed (Figure 6d,e). GalNAc‐PRX on the surface of nanoparticles lowered the ζ‐potentials derived from 5G/RNP by hindering the cationic surface of the complex (Figure 6e). This neutralization might have inhibited the aggregation of unit (or lower order) particles into secondary (or higher order) particles, leading to the compaction of the particle sizes (Figure 6d). These results suggest that GalNAc‐PRX was successfully functionalized on the surface of 5G/RNP.

To investigate the effect of GalNAc‐PRX functionalization, the intracellular uptakes of GalNAc‐PRX/5G/RNP and 5G/RNP were compared (Figure 6f–h). In the absence of serum, 5G/RNP showed significantly higher efficacy of intracellular uptake than GalNAc‐PRX/5G/Cas9 RNP (Figure 6f). In contrast, GalNAc‐PRX/5G/RNP showed markedly greater cellular internalization than 5G/RNP in the presence of serum (Figure 6g). In addition, the intracellular uptake of 5G/RNP was reduced in the presence of serum, whereas that of GalNAc‐PRX/5G/RNP remained (Figure 6h) because of the neutralization of the cationic surface of 5G/RNP and the inhibition of serum‐derived protein absorption. In other words, GalNAc‐PRX functionalization could inhibit non‐specific internalization by the cation of 5G/RNP and promote specific ASGPR‐mediated endocytosis (Figure 6i). These results suggest that GalNAc‐PRX may be useful for the surface functionalization of cationic nanoparticles to endow the stability and targeting ability to hepatocytes.

Inspired by these results, we next evaluated GalNAc‐PRX/5G/RNP in vivo (Figure 6j–l). After a single intravenous administration, GalNAc‐PRX/5G/RNP showed an ∼25% lowering effect of targeted protein encoded targeted gene, i.e., transthyretin (TTR), which is a promising therapeutic target for hereditary TTR amyloidosis. Additionally, the genome‐editing efficacy of GalNAc‐PRX/5G/RNP was significantly higher than that of 5G/RNP. Observations based on an In Vivo Imaging System (IVIS) showed that GalNAc‐PRX enhanced the hepatic distribution of fluorescent‐labeled RNP after the systemic administration. These results suggest that GalNAc‐PRX/5G has the potential as a novel liver‐targeting delivery system for Cas9 RNP, thus facilitating the development of therapeutic agents based on CRISPR‐Cas9 genome editing.

In this study, we focused on the functionalization of *mono*GalNAc with a PRX backbone, which is synthesized by established methodologies that ensure high yield and homogeneity [25, 26, 27]. While our findings suggest that *mono*GalNAc‐modified PRX, which has a fixed M.W. and backbone (αCD/PEG) serves as a facile and efficient liver‐targeting strategy, the system could be further optimized by systematically investigating the impact of polymer length, coverage, and interaction of cyclic molecules and an axile molecule. Determining the minimum effective polymer length required for optimal performance would contribute to the development of more streamlined and sophisticated delivery architectures. Furthermore, CD coverage remains a critical factor to be explored, as it directly attributes the molecular mobility of the CD units along the axile PEG.

Regarding characterization in this study, although mechanical interlocked structure of PRXs was verified using 2D‐NMR (NOESY) (Figures S7, S10, S21, and S29) and thin layer chromatography as well as purity (Figure S33), the development and optimization of novel PRX backbones or the evaluation of synthesized compounds would benefit from higher‐resolution analytical techniques, such as high‐resolution mass spectrometry and size‐exclusion chromatography [50, 51]. Additionally, the application of quasi‐elastic neutron scattering [32] to directly quantify the sliding motion of CDs on the PRX could enable more precise tuning of molecular mobilities. Nevertheless, this work highlights the significant potential of leveraging PRX molecular mobility to achieve highly efficient targeting with simple *mono*GalNAc units, offering a promising alternative to conventional *tri*GalNAc‐based strategies.

## 3 Conclusion

In this study, an alternative and superior strategy for targeting ASGPRs by *mono*GalNAc modified in the CDs of PRX was developed. *Mono*GalNAc‐PRX was prepared using a much simpler process than that required by conventional *tri*GalNAc. Comparative studies of synthesized GalNAc‐modified polymers suggested that the inherent molecular mobility of CDs on PRX enabled highly efficient and facile multivalent interactions between modified *mono*GalNAcs and ASGPRs, which is might have been enabled by adjusting the presentation of modified GalNAcs to ASGPRs without any spatial mismatches and clustering of *mono*GalNAcs in a trivalent‐like manner. Furthermore, *mono*GalNAc‐PRX‐LYTAC and *mono*GalNAc‐PRX‐functionalized genome‐editing nanoparticles are highly promising alternatives to conventional *tri*GalNAc‐based drug delivery systems. The results obtained in this study provide valuable insights into the development of GalNAc ligands and may facilitate the development of more robust and highly potent liver‐targeting drug delivery systems.

## 4 Experimental Section

### 4.1 General

All chemicals and reagents used in this study are listed in Table S5. All the chemicals and solvents were of analytical grade. Unless otherwise noted, deionized water was used throughout the study. The sequences of non‐targeted control sgRNA (sgControl) and GFP‐targeted sgRNA (sgGFP) are available in our previous report [35, 37]. Murine transthyretin (TTR)‐targeted sgRNA has been reported by Finn et al. [52].

### 4.2 Preparation of Materials

#### 4.2.1 *N*‐Acetyl Galactosamine (GalNAc)‐Spacer‐NH_2_

The precursors of GalNAc‐spacer‐NH_2_, oxazoline derivative, and chloroethylene glycol derivative were prepared using the method reported by Pujol et al. [53] with some modifications. Galactosamine pentaacetate (2.4 g, 6.16 mmol) was dispersed in dry dichloromethane (20 mL). Molecular sieves (4 Å) and trimethylsilyl trifluoromethanesulfonate (TMSOTf, 3.78 mL, 21 mmol) were added, and the reaction mixture was stirred under a nitrogen atmosphere for 18 h at 50°C with reflux condition. Next, the reaction was quenched by adding triethylamine (1.6 mL) on ice. Subsequently, the mixture was diluted with dichloromethane (200 mL), washed with saturated aqueous sodium bicarbonate (200 mL) and water (200 mL), evaporated, and dried under reduced pressure. The resulting crude oxazoline was dissolved in dry dichloromethane (34 mL). Next, molecular sieves (4 Å) and 2‐[2‐(2‐chloroethoxy)ethoxy]ethanol (1.38 mL, 9.5 mmol) were added to the reaction mixture. Subsequently, TMSOTf (0.62 mL, 3.42 mmol) was added, and the reaction mixture was stirred under a nitrogen atmosphere for 18 h at room temperature. The reaction was quenched by adding triethylamine (1.6 mL) on ice. The mixture was diluted with dichloromethane (300 mL) and washed with saturated aqueous sodium bicarbonate (200 mL) and water (200 mL). The resulting organic phase was centrifuged, filtered, evaporated, and dried at reduced pressure.

The crude chloroethylene glycol derivative was dissolved in dry dimethyl sulfoxide (DMSO, 10 mL). The reaction mixture was added dropwise to excess ethylenediamine (EDA, 8.0 mL, 61.16 mmol) at 50°C under stirring. Subsequently, it was stirred for 18 h at 50°C and centrifuged. The resulting supernatant was added to cold diethyl ether and centrifuged. The precipitate was washed several times with cold diethyl ether and a diethyl ether/acetone mixture. The resulting precipitate was dissolved in water and evaporated. The process of re‐dissolution and concentration by evaporation was repeated at least five times to completely remove any unreacted ethylenediamine. The resulting concentrated solution was lyophilized to obtain (Ac)GalNAc‐spacer‐NH_2_ (yield: 879.6 mg, 36%).

^1^H NMR (400 MHz, DMSO‐*d*_6_, δ): 4.25 (d, *J* = 7.6 Hz, 1H; ─N **H** Ac), 4.25 (d, *J* = 4.2 Hz, 1H; H1 of GalNAc), 3.98–2.94 (m, 16H + HDO; H2, H3, H4, H5, 2 × H6 of GalNAc + ─OC **H 2** C **H 2** OC **H 2** C **H 2** OC **H 2** ─), 2.83–2.55 (br m, 6H; ─C **H 2** NHC **H 2** C **H 2** NH_2_), 1.83 (s, 0, 3, 6 or 9H;─OCOC **H 3**), 1.78 (s, 3H; ─NHCOC **H 3**); ^13^C NMR (125 MHz, DMSO‐*d*_6_, δ): 169.53 (**C** ═O), 101.39 (C1 of GalNAc), 75.27 (C5 of GalNAc), 71.51, 69.89, 69.71, 69.60, 69.57, 67.55, 67.39 (C3, C4, and C6 of GalNAc + ─O **C** H_2_ **C** H_2_O **C** H_2_ **C** H_2_O **C** H_2_ **C** H_2_NH─), 60.34 (C2 of GalNAc), 51.94 (─NH **C** H_2_CH_2_NH_2_),48.09, 47.34 (─ **C** H_2_NH **C** H_2_─), 40.64 (─ **C** H_2_NH_2_), 23.03 (─NHCO **C** H_3_) 22.59 (─OCO **C** H_3_); FAB‐MS *m*/*z*: [M + H]^+^ calcd for C_18_H_36_N_3_O_9_9, 396.2; found, 396.4.

#### 4.2.2 Fluorescein‐Capped Polyrotaxane (FAM‐PRX)

Polyethylene glycol (PEG)‐diamino terminated (DAT) was prepared based on the method reported by Araki et al. [27]. To obtain polypseudorotaxanes (PpRX), PEG‐DAT (20 kDa, 3.0 g) was added to a 12% (w/v) α‐cyclodextrin (αCD) aqueous solution (100 mL) and stirred overnight at 4°C. The precipitates were obtained via centrifugation and then dried via lyophilization.

5(6)‐Carboxylfluorescein (FAM‐COOH, 340 mg, 0.9 mmol), benzotriazol‐1‐yloxytris(dimethylamino)phosphonium hexafluorophosphate (BOP) reagent (375 mg, 0.84 mmol), and *N*‐ethyldiisopropylamine (EDIPA, 0.163 mL, 0.96 mmol) were dissolved in dimethylformamide (DMF, 7.14 mL), and the αCD/PEG‐DAT (20 kDa) PpRX (1.0 g) was added. After stirring overnight at 4°C, the precipitate was collected by centrifugation and washed twice with methanol/DMF (1:1, v/v) and twice with methanol. The resulting precipitate was dissolved in DMSO and dialyzed against water for 3 days (molecular weight cutoff (MWCO): 6–8 kDa). The precipitate in a dialysis back was obtained via centrifugation and washed five times with acetone and water, respectively. The resulting product was lyophilized to obtain FAM‐PRX (yield: 666 mg, 90% (based on PEG)).

^1^H NMR (400 MHz, DMSO‐*d*_6_, δ): 6.67–6.51 (br m, aromatic proton of FAM), 5.60 (br s, ─O(2) **H** of αCD), 5.44 (br s, ─O(3) **H** of αCD), 4.76 (br s, H1 of αCD), 4.40 (br s, ─O(6) **H** of αCD), 3.79–3.11 (br m, H2–5, 2 × H6 of αCD, *n* × ─C **H 2** C **H 2** OC **H 2** C **H 2** O─ of PEG); ^13^C NMR (125 MHz, DMSO‐*d*_6_, δ): 101.95 (C1 of αCD), 81.71 (C4 of αCD), 73.35, 72.12, 71.59 (C2, C3, C5 of αCD), 69.74, 69.48 (*n* × ─ **C** H_2_ **C** H_2_O **C** H_2_ **C** H_2_O─ of PEG), 59.50 (C6 of αCD).

#### 4.2.3 Hydroxypropyl FAM‐PRX (FAM‐HP‐PRX)

FAM‐PRX (250 mg) was dissolved in a 1N sodium hydroxide aqueous solution (50 mL). Propylene oxide (9.0 mL) was added dropwise to the FAM‐PRX solution with stirring on ice. The reaction mixture was stirred overnight on ice, dialyzed against water for 3 days (MWCO: 25 kDa), and lyophilized to obtain FAM‐HP‐PRX (yield: 259 mg, 96%).

^1^H NMR (400 MHz, D_2_O, δ): 6.81–6.61 (br m, aromatic proton of FAM), 5.16–4.81 (br m, H1 of αCD), 4.03–3.10 (br m, H2–5, 2 × H6 of αCD, *n* × ─C **H 2** C **H 2** OC **H 2** C **H 2** O─ of PEG, ─C **H 2** C **H** ─ of HP group), 1.08–0.93 (br m, ─C **H 3** of HP group); ^13^C NMR (125 MHz, DMSO‐*d*_6_, δ): 103.21, 102.03, 100.00 (C1 of αCD), 81.20 (C4 of αCD), 76.50 (─ **C** H_2_─ of HP group),73.75, 72.06, 71.84 (C2, C3, C5 of αCD), 69.85, 69.64 (*n* × ─ **C** H_2_ **C** H_2_O **C** H_2_ **C** H_2_O─ of PEG), 66.35 (─CH─ of HP group) 59.97 (C6 of αCD), 18.50 (─ **C** H3 of HP group). The number of modified HP group/αCD, the coverage (%) of αCD to PEG, and No. of αCD/PRX were calculated by the integral value (*I*) of ^1^H NMR spectrum; 2 × *I_3_*/*I_1_*, 4 ×*I_1_*/(*I_2_* – 6 × *I_1_* – *I_3_*)/3 × 100, 20 000/88 × coverage (%)/100, respectively. *I_1_* = 5.16–4.81 (H1 of αCD). *I_2_* = 4.03–3.10 (H2–5, 2 × H6 of αCD, *n* × ─C **H 2** C **H 2** OC **H 2** C **H 2** O─ of PEG, ─C **H 2** C **H** ─ of HP group). *I_3_* = 1.08–0.93 ppm (─C **H 3** of HP group).

#### 4.2.4 Hydroxypropyl FAM‐Dextran (FAM‐HP‐DEX)

To obtain EDA‐DEX, dextran 70 (700 mg, 0.01 mmol) was dissolved in dry DMSO (20 mL), and *N,N*‐carbonyldiimidazole (CDI, 20 mg, 0.124 mmol) was added to the solution. The reaction mixture was stirred overnight at 25°C under a nitrogen atmosphere, added dropwise to EDA (83 µL, 1.24 mmol), and stirred overnight at room temperature under a nitrogen atmosphere. Subsequently, the reaction mixture was dialyzed against water for 3 days (MWCO: 6–8 kDa) and dried via lyophilization.

FAM‐COOH (27 mg, 70.8 µmol) and *N*‐hydroxysuccinimide (NHS, 16 mg, 141.6 µmol) were dissolved in dry DMSO (1.0 mL). Next, 1‐(3‐Dimethylaminopropyl)‐3‐ethylcarbodiimide hydrochloride (EDC, 27 mg, 141.6 µmol) was added and stirred for 4 h at room temperature to activate the carboxylic acid group of FAM‐COOH. Subsequently, the FAM‐NHS‐ester solution was added dropwise to EDA‐DEX (400 mg, 5.72 µmol) and then dissolved in dry DMSO (5.7 mL). The reaction mixture was stirred overnight at room temperature, dialyzed against water, and lyophilized to obtain FAM‐DEX.

FAM‐DEX (215 mg) was dissolved in 1N sodium hydroxide aqueous solution (43 mL). Propylene oxide (7.74 mL) was added dropwise to the FAM‐DEX solution with stirring on ice. The reaction mixture was stirred overnight on ice, dialyzed against water for 3 days (MWCO: 25 kDa), and lyophilized to obtain FAM‐HP‐DEX. The fluorescence intensities of FAM‐HP‐PRX and FAM‐HP‐DEX were confirmed to be almost identical (yield: 251 mg, 87%).

^1^H NMR (400 MHz, D_2_O, δ): 6.75–6.51 (br m, aromatic proton of FAM), 5.14–4.72 (br m, H1 of DEX), 4.04–3.08 (br m, H2–5, 2 × H6 of DEX, ─C **H 2** C **H** ─ of HP group), 1.21–0.76 (br m, ─C **H 3** of HP group). The number of modified HP group/DEX was calculated by the integral value (*I*) of ^1^H NMR spectrum; *I_2_*/(3 × *I_1_)* × No. of glucose unit of DEX. *I_1_* = 5.14–4.72 (H1 of DEX). *I_2_* = 1.21–0.76 ppm (─C **H 3** of HP group).

#### 4.2.5 Monovalent GalNAc‐Modified FAM‐HP‐PRX (*Mono*GalNAc‐PRX)

FAM‐HP‐PRX (20 mg, 249 nmol, αCD: 12.2 µmol) was dissolved in dry DMSO (1.2 mL), and CDI (21.6 mg, 134.4 µmol) was added to the solution. The reaction mixture was stirred for 6 h at 25°C under a nitrogen atmosphere, added dropwise to a dry DMSO solution (2 mL) containing (Ac)GalNAc‐spacer NH_2_ (160 mg, 403 µmol), and stirred 6 h at room temperature under a nitrogen atmosphere. Subsequently, the reaction mixture was dialyzed against water for 1 day (MWCO: 25 kDa), and the solution in the dialysis bag was collected. An aqueous solution of sodium hydroxide (10N) was added to the collected solution on ice to adjust the concentration of sodium hydroxide at 1N. The solution was stirred for 2 h on ice, dialyzed against water for another 2 days (MWCO: 25 kDa), and lyophilized to obtain *mono*GalNAc‐PRX (yield: 19.2 mg, 77%).

^1^H NMR (400 MHz, D_2_O, δ): 6.56–6.44 (br m, aromatic proton of FAM), 5.32–4.76 (br m, H1 of αCD), 4.48–4.16 (br m, H1 of GalNAc), 4.12–2.58 (br m, H2–5, 2 × H6 of αCD, H2–5, 2 × H6 of GalNAc, *n* × ─C **H 2** C **H 2** OC **H 2** C **H 2** O─ of PEG, ─OC **H 2** C **H 2** OC **H 2** C **H 2** OC **H 2** ─,─C **H 2** NHCO─, ─C **H 2** NHC **H 2** ─ of GalNAc‐spacer, ─C **H 2** C **H** ─ of HP group), 2.19–1.60 (br m, ─COC **H 3** of GalNAc), 1.25–0.53 (br m, ─C **H 3** of HP group). The number of modified GalNAc/αCD was calculated by the integral value (*I*) of ^1^H NMR spectrum; 6 × *I_2_*/*I_1_*. *I_1_* = 5.16–4.81 (H1 of αCD). *I_2_* = 4.48–4.24 (H1 of GalNAc).

#### 4.2.6 Triantennary GalNAc‐Modified FAM‐HP‐PRX (*tri*GalNAc‐PRX)

FAM‐HP‐PRX (20 mg, 249 nmol, αCD: 12.2 µmol) was dissolved in dry DMSO (1.2 mL), and CDI (15.6 mg, 96.72 µmol) was added to the solution and stirred overnight at 25°C under a nitrogen atmosphere. The dry DMSO solution (1 mL) containing Tris[[2‐(tert‐butoxycarbonyl)ethoxy]methyl]methylamine (147 mg, 0.29 mmol) was added dropwise to the reaction mixture and stirred overnight at 25°C under a nitrogen atmosphere. Subsequently, the reaction mixture was dialyzed against DMSO for 2 h (MWCO: 25 kDa), and the solution in the dialysis bag was collected. Trifluoroacetic acid (1.0 mL) was added to the collected solution, stirred for 1 h, dialyzed against DMSO for 2 h (MWCO: 25 kDa), and evaporated. The evaporated solution was dialyzed against water for another 2 days (MWCO: 25 kDa) and lyophilized.

The resulting PRX (20 mg) and NHS (8.0 mg, 67 µmol) were dissolved in dry DMSO (2.0 mL). Next, EDC (13 mg, 67 µmol) was added and stirred for 3 h at 25°C. Subsequently, the reaction mixture was added dropwise to a dry DMSO solution (2.0 mL) containing (Ac)GalNAc‐spacer‐NH_2_ (160 mg, 403 µmol) and triethylamine (28 µL, 202 µmol) and stirred for 6 h at 25°C. Subsequently, the reaction mixture was dialyzed against water for 1 day (MWCO: 25 kDa), and the solution in the dialysis bag was collected. An aqueous solution of sodium hydroxide (10N) was added to the collected solution on ice to adjust the concentration of sodium hydroxide at 1N. The solution was stirred for 2 h on ice, dialyzed against water for another 2 days (MWCO: 25 kDa), and lyophilized to obtain *tri*GalNAc‐PRX (yield: 18.8 mg, 79%).

^1^H NMR (400 MHz, D_2_O, δ): 6.56–6.44 (br m, aromatic proton of FAM), 5.32–4.76 (br m, H1 of αCD), 4.48–4.16 (br m, H1 of GalNAc), 4.12–2.76 (br m, H2–5, 2 × H6 of αCD, H2–5, 2 × H6 of GalNAc, *n* × ─C **H 2** C **H 2** OC **H 2** C **H 2** O─ of PEG, ─OC **H 2** C **H 2** OC **H 2** C **H 2** OC **H 2** ─, ─C **H 2** NHCO─, ─C **H 2** NHC **H 2** ─, ─C **H 2** OC **H 2** ─ of *tri*GalNAc, ─C **H 2** C **H** ─ of HP group), 2.76–2.23 (br m, ─NHCOC **H 2** of *tri*GalNAc), 2.19–1.60 (br m, ─COC **H 3** of GalNAc), 1.36–0.64 (br m, ─C **H 3** of HP group). The number of modified *tri*GalNAc/PRX was calculated by the integral value (*I*) of ^1^H NMR spectrum; 6/3 × *I_2_*/*I_1_* × No. of αCD. *I_1_* = 5.23–4.74 (H1 of αCD). *I_2_* = 4.39–4.27 (H1 of GalNAc).

#### 4.2.7 GalNAc‐Modified Immobile Control Polymers (*Mono*GalNAc‐DEX and *tri*GalNAc‐DEX)

*Mono*GalNAc‐DEX and *tri*GalNAc‐DEX were prepared in accordance with the procedures described above for *mono*GalNAc‐PRX and *tri*GalNAc‐PRX, however, FAM‐HP‐DEX was used.

Briefly, to obtain *mono*GalNAc‐DEX, FAM‐HP‐DEX (20 mg, 213 nmol) was activated by CDI (18.5 mg, 115 µmol) and reacted with (Ac)GalNAc‐spacer‐NH_2_ (136 mg, 345 µmol). Purification and *O*‐deacetylation were performed as described previously (yield: 18.7 mg, 78%).

^1^H NMR (400 MHz, D_2_O, δ): 6.61–6.43 (br m, aromatic proton of FAM), 5.26–4.70 (br m, H1 of DEX), 4.39–4.26 (br m, H1 of GalNAc), 4.24–2.50 (br m, H2–5, 2 × H6 of DEX, H2–5, 2 × H6 of GalNAc, ─OC **H 2** C **H 2** OC **H 2** C **H 2** OC **H 2** ─,─C **H 2** NHCO─, ─C **H 2** NHC **H 2** ─ of GalNAc‐spacer, ─C **H 2** C **H** ─ of HP group), 1.99–1.72 (br m, ─COC **H 3** of GalNAc), 1.25–0.61 (br m, ─C **H 3** of HP group); The number of modified GalNAc/DEX was calculated by the integral value (*I*) of ^1^H NMR spectrum; *I_2_*/*I_1_* × No. of glucose unit of DEX. *I_1_* = 5.15–4.70 (H1 of DEX). *I_2_* = 4.40–4.26 (H1 of GalNAc).

To obtain *tri*GalNAc‐DEX, FAM‐HP‐DEX was activated by CDI (13.3 mg, 92.99 µmol) and reacted with tris[[2‐(tert‐butoxycarbonyl)ethoxy]methyl]methylamine (125 mg, 0.25 mmol). Purification and deprotection of tertiary butyl groups were conducted as described above. The resulting DEX was activated by NHS (7.0 mg, 58 µmol) and EDC (12 mg, 58 µmol) and reacted with (Ac)GalNAc‐spacer‐NH_2_ (136 mg, 345 µmol) with triethylamine (24 µL, 172.5 µmol). Purification and *O*‐deacetylation were performed as described previously (yield: 16.5 mg, 71%).

^1^H NMR (400 MHz, D_2_O, δ): 6.61–6.43 (br m, aromatic proton of FAM), 5.26–4.70 (br m, H1 of DEX), 4.39–4.26 (br m, H1 of GalNAc), 4.24–2.67 (br m, H2–5, 2 × H6 of DEX, H2–5, 2 × H6 of GalNAc, ─OC **H 2** C **H 2** OC **H 2** C **H 2** OC **H 2** ─, ─C **H 2** NHCO─, ─C **H 2** NHC **H 2** ─, ─C **H 2** OC **H 2** ─ of *tri*GalNAc, ─C **H 2** C **H** ─ of HP group), 2.66–2.24 (br m, ─NHCOC **H 2** of *tri*GalNAc), 1.99–1.72 (br m, ─COC **H 3** of GalNAc), 1.25–0.61 (br m, ─C **H 3** of HP group). The number of modified *tri*GalNAc/DEX was calculated by the integral value (*I*) of ^1^H NMR spectrum; 1/3 × *I_2_*/*I_1_* × No. of glucose unit of DEX. *I_1_* = 5.22–4.69 (H1 of DEX). *I_2_* = 4.40–4.29 (H1 of GalNAc).

#### 4.2.8 *Mono*GalNAc‐Modified Dibenzocyclooctyne (DBCO)‐Capped HP‐PRX (DBCO‐GalNAc‐PRX)

To obtain carboxylic acid‐end adamantane (Ad) capped PRX (HOOC‐Ad‐PRX), 1,3‐adamantanediacetic acid (597 mg, 2.4 mmol), BOP reagent (656 mg, 1.47 mmol), and EDIPA (0.285 mL, 1.65 mmol) were dissolved in DMF (7.14 mL); αCD/PEG‐DAT (20 kDa) PpRX (1.0 g) was added, and the mixture was stirred overnight at room temperature. Purification was performed based on the method for FAM‐PRX (yield: 572 mg, 80% (based on PEG)).

^1^H NMR (600 MHz, DMSO‐*d*_6_, δ): 5.62 (br s, ─O(2) **H** of αCD), 5.46 (br s, ─O(3) **H** of αCD), 4.78 (br s, H1 of αCD), 4.41 (br s, ─O(6) **H** of αCD), 3.79–3.23 (br m, H2–5, 2 × H6 of αCD, *n* × ─C **H 2** C **H 2** OC **H 2** C **H 2** O─ of PEG).

HOOC‐Ad‐PRX (100 mg) was dissolved in 1N sodium hydroxide aqueous solution (20 mL). Propylene oxide (3.61 mL) was added dropwise to the HOOC‐Ad‐PRX solution with stirring on ice. The reaction mixture was stirred overnight on ice, dialyzed against water for 3 days (MWCO: 50 kDa), and lyophilized to obtain HOOC‐Ad‐HP‐PRX (yield: 102 mg, 94%).

^1^H NMR (500 MHz, D_2_O, δ): 5.16–4.83 (br m, H1 of αCD), 4.09–3.03 (br m, H2–5, 2 × H6 of αCD, *n* × ─C **H 2** C **H 2** OC **H 2** C **H 2** O─ of PEG, ─C **H 2** C **H** ─ of HP group), 1.21–0.62 (br m, ─C **H 3** of HP group). The number of modified HP group/αCD, the coverage (%) of αCD to PEG, and No. of αCD/PRX were calculated by the integral value (*I*) of ^1^H NMR spectrum; 2 × *I_3_*/*I_1_*, 4 ×*I_1_*/(*I_2_* – 6 × *I_1_* – *I_3_*)/3 × 100, 20 000/88 × coverage (%)/100, respectively. *I_1_* = 5.16–4.81 (H1 of αCD). *I_2_* = 4.03–3.10 (H2–5, 2 × H6 of αCD, *n* × ─C **H 2** C **H 2** OC **H 2** C **H 2** O─ of PEG, ─C **H 2** C **H** ─ of HP group). *I_3_* = 1.08–0.93 ppm (─C **H 3** of HP group).

To prepare DBCO‐HP‐PRX, HOOC‐Ad‐HP‐PRX (20 mg, 215 nmol), BOP reagent (2.49 mg, 6.45 µmol), EDIPA (1.2 µL, 6.45 µmol), and DBCO‐amine (1.8 mg, 6.45 µmol) were dissolved in dry DMSO (0.6 mL) and stirred overnight at room temperature. The reaction mixture was added dropwise to cold acetone and the resulting precipitate was collected via centrifugation, washed five times with cold acetone, dissolved in water, and lyophilized (yield: 16.5 mg, 81.3%).

^1^H NMR (500 MHz, D_2_O, δ): 7.49–6.85 (br m, aromatic proton of DBCO), 5.24–4.82 (br m, H1 of αCD), 4.17–2.96 (br m, H2–5, 2 × H6 of αCD, *n* × ─C **H 2** C **H 2** OC **H 2** C **H 2** O─ of PEG, ─C **H 2** C **H** ─ of HP group), 1.17–0.88 (br m, ─C **H 3** of HP group).

DBCO‐HP‐PRX (10 mg, 106 nmol, αCD: 6.68 µmol) was dissolved in dry DMSO (0.6 mL), and CDI (16.1 mg, 99 µmol) was added to the solution. The reaction mixture was stirred overnight at 25°C under a nitrogen atmosphere, added dropwise to a dry DMSO solution (0.5 mL) containing (Ac)GalNAc‐spacer NH_2_ (101 mg, 254 µmol), and stirred for 6 h at 25°C under a nitrogen atmosphere. Subsequently, the reaction mixture was dialyzed against water for 2 days (MWCO: 50 kDa) and lyophilized to obtain DBCO‐GalNAc‐PRX (yield: 12.0 mg, 84%).

^1^H NMR (500 MHz, D_2_O, δ): 7.70–7.28 (br m, aromatic proton of DBCO), 5.26–4.85 (br m, H1 of αCD), 4.45–4.13 (br m, H1 of GalNAc), 4.13–2.50 (br m, H2–5, 2 × H6 of αCD, H2–5, 2 × H6 of GalNAc, *n* × ─C **H 2** C **H 2** OC **H 2** C **H 2** O─ of PEG, ─OC **H 2** C **H 2** OC **H 2** C **H 2** OC **H 2** ─,─C **H 2** NHCO─, ─C **H 2** NHC **H 2** ─ of GalNAc‐spacer, ─C **H 2** C **H** ─ of HP group), 1.98–1.68 (br m, ─COC **H 3** of GalNAc), 1.31–0.64 (br m, ─C **H 3** of HP group). The number of modified GalNAc/αCD was calculated by the integral value (*I*) of ^1^H NMR spectrum; 6 × *I_2_*/*I_1_*. *I_1_* = 5.16–4.81 (H1 of αCD). *I_2_* = 4.48–4.24 (H1 of GalNAc).

#### 4.2.9 Fc‐Specific Azide Modification of Antibody

To obtain the N_3_‐antibody, azide modification of the anti‐bovine serum albumin (BSA) antibody was conducted using the Siteclick Antibody Azido Modification Kit. Briefly, anti‐BSA was treated with β‐galactosidase to digest the carbohydrate group of the Fc region of the antibody. Subsequently, the antibody was incubated with galactose‐1‐phospahte uridylyltransferase (GalT) and UDP‐*N*‐azidoacetylgalactosamine disodium (UDP‐GalNAz) to introduce an azide‐functionalized sugar to the digested end of the carbohydrate group in the Fc region of the antibody.

#### 4.2.10 Lysosome‐Targeting Antibody Chimeras (LYTACs) via Click Chemistry

To obtain the N_3_‐antibody, azide modification of the anti‐bovine serum albumin (BSA) antibody was conducted using the Siteclick Antibody Azido Modification Kit. Briefly, anti‐BSA was treated with β‐galactosidase to digest the carbohydrate group of the Fc region of the antibody. Subsequently, the antibody was incubated with galactose‐1‐phospahte uridylyltransferase (GalT) and UDP‐*N*‐azidoacetylgalactosamine disodium (UDP‐GalNAz) to introduce an azide‐functionalized sugar to the digested end of the carbohydrate group in the Fc region of the antibody.

#### 4.2.11 *Mono*GalNAc‐Modified Ad‐Capped HP‐PRX (Ad‐cap‐GalNAc‐PRX)

Ad‐capped PRX (Ad‐cap‐PRX) was prepared based on our previous report [35, 36, 37] using the αCD/PEG‐DAT (35 kDa) PpRX and 1‐adamantane acetic acid. HP modification of Ad‐cap‐PRX was performed as described above. Ad‐cap‐HP‐PRX (20 mg, 136 nmol, αCD: 11.6 µmol) was dissolved in dry DMSO (1.2 mL), and CDI (16 mg, 98.5 µmol) was added to the solution. The reaction mixture was stirred overnight at 25°C under a nitrogen atmosphere, added dropwise to a dry DMSO solution (1.0 mL) containing (Ac)GalNAc‐spacer NH_2_ (117 mg, 295 µmol), and stirred for 6 h at 25°C under a nitrogen atmosphere. Purification and *O*‐deacetylation were performed as described previously (yield: 19.6 mg, 70%).

^1^H NMR (500 MHz, D_2_O, δ): 5.18–4.89 (br m, H1 of αCD), 4.48–4.21 (br m, H1 of GalNAc), 4.21–2.44 (br m, H2–5, 2 × H6 of αCD, H2–5, 2 × H6 of GalNAc, *n* × ─C **H 2** C **H 2** OC **H 2** C **H 2** O─ of PEG, ─OC **H 2** C **H 2** OC **H 2** C **H 2** OC **H 2** ─,─C **H 2** NHCO─, ─C **H 2** NHC **H 2** ─ of GalNAc‐spacer, ─C **H 2** C **H** ─ of HP group), 2.05–1.77 (br m, ─COC **H 3** of GalNAc), 1.32–0.51 (br m, ─C **H 3** of HP group).The number of modified GalNAc/αCD was calculated by the integral value (*I*) of ^1^H NMR spectrum; 6 × *I_2_*/*I_1_*. *I_1_* = 5.16–4.81 (H1 of αCD). *I_2_* = 4.48–4.24 (H1 of GalNAc).

#### 4.2.12 BetaCD‐Capped Aminated PRX 5th Generation (βCD‐cap‐amino‐PRX (5G))

To obtain βCD‐capped PRX (βCD‐cap‐PRX), 6‐O‐α‐(4‐O‐α‐D‐Glucuronyl)‐D‐glucosyl‐β‐CD (GUGβCD, 1.35 g, 0.9 mmol), BOP reagent (375 mg, 0.84 mmol), and EDIPA (0.163 mL, 0.94 mmol) were dissolved in DMF (28.57 mL), and the αCD/PEG‐DAT (35 kDa) PpRX (1.0 g) was added and stirred overnight at room temperature. Purification was performed based on the method for FAM‐PRX (yield: 444 mg, 59% (based on PEG)).

^1^H NMR (600 MHz, DMSO‐*d*_6_, δ): 5.61 (br s, ─O(2) **H** of αCD and GUGβCD), 5.46 (br s, ─O(3) **H** of αCD and GUGβCD), 4.76 (br s, H1 of αCD and GUGβCD), 4.41 (br s, ─O(6) **H** of αCD and GUGβCD), 3.79–3.23 (br m, H2–5, 2 × H6 of αCD and GUGβCD, *n* × ─C **H 2** C **H 2** OC **H 2** C **H 2** O─ of PEG). The coverage (%) of αCD to PEG and No. of αCD/PRX were calculated by the integral value (*I*) of ^1^H NMR spectrum; 4 × [*I_1_* – 14 × *I_2_*/(4 ×35 000/44)]/(3 × *I_2_*) × 100, 35 000/88 × coverage (%)/100, respectively. *I_1_* = 5.61 (H1 of αCD and GUGβCD). *I_2_* = 3.58–3.45 (*n* × ─C **H 2** C **H 2** OC **H 2** C **H 2** O─ of PEG).

The amination of βCD‐cap‐PRX to βCD‐cap‐amino‐PRX (5G) was conducted based on our previous report [35, 36, 37], however using βCD‐cap‐PRX.

^1^H NMR (600 MHz, D_2_O, δ): 5.18–4.93 (br m, H1 of αCD), 4.02–3.03 (br m, H2–5, 2 × H6 of αCD, *n* × ─C **H 2** C **H 2** OC **H 2** C **H 2** O─ of PEG, ─OC **H 2** C **H 2** OC **H 2** C **H 2** OC **H 2** ─,─C **H 2** NHCO─, ─CONHC **H 2** ─ of modified cystamine and diethylenetriamine group), 3.03–2.83 (br m, ─C **H 2** SSC **H 2** C **H 2** NH_2_ of cystamine), 2.83–2.61 (br m, ─C **H 2** NHC **H 2** C **H 2** NH_2_ of diethylenetriamine). The number of modified cystamine/αCD and diethylenetriamine /αCD was calculated by the integral value (*I*) of ^1^H NMR spectrum; *I_2_*/*I_1_* and *I_3_*/*I_1_*, respectively. *I_1_* = 5.18–4.93 (H1 of αCD). *I_2_* = 3.03–2.83 (─C **H 2** SSC **H 2** C **H 2** NH_2_ of cystamine). *I_3_* = 2.83–2.61 (─C **H 2** NHC **H 2** C **H 2** NH_2_ of diethylenetriamine).

#### 4.2.13 GalNAc‐PRX/amino‐PRX (5G)/Cas9 RNP Complexes

Ad‐cap‐GalNAc‐PRX was dissolved in Hank's balanced salt solution (HBSS). The solid βCD‐cap‐amino‐PRX (5G) was added to the Ad‐cap‐GalNAc‐PRX solution and vortexed to dissolve βCD‐cap‐amino‐PRX (5G) and to prepare an A (Ad‐cap‐GalNAc‐PRX)/B (βCD‐cap‐amino‐PRX (5G))/A‐type pseudo block copolymer via host‐guest interactions. The molar ratio between Ad‐cap‐GalNAc‐PRX and βCD‐cap‐amino‐PRX (5G) was set at 2:1. The final concentration of βCD‐cap‐amino‐PRX (5G) in stock solution was set to 1.0 and 2.0 mg/mL for in vitro and in vivo studies, respectively.

Recombinant Cas9 protein (N‐terminal modified with NLS) and sgRNA were mixed and incubated in nuclease‐free water or Opti‐MEM at a 1:1 molar ratio for 10 min at room temperature to obtain Cas9 RNP. Subsequently, the Cas9 RNP solution was added to the Ad‐cap‐GalNAc‐PRX/βCD‐cap‐amino‐PRX (5G)/Ad‐cap‐GalNAc‐PRX solution, gently pipetted, and incubated for 15 min at room temperature. The amount of polymer was set to 125% of imprinting to Cas9 RNP. The solution was diluted with the optimal solvents for further in vitro or in vivo assays. To confirm the complex formation, Cas9 RNP complexes (sgRNA: 1.0 µg) were diluted in 1.0 mL of HBSS (pH 7.4), and the particle sizes and ζ‐potentials of Cas9 RNP were measured by using a Zetasizer Pro apparatus (Malvern Instruments, Worcestershire, UK).

### 4.3 Cell Culture

A human liver cancer cell line (HepG2) and a human cervical epithelioid carcinoma cell line (HeLa) were obtained from Riken Bioresource Center (Tsukuba, Japan) and cultured in Dulbecco's modified Eagle's medium (DMEM, high glucose) containing glutamine (2 mm), penicillin (100 U mL^−1^), and streptomycin (100 mg L^−1^) with 10% fetal bovine serum (FBS) at 37°C in a humidified 5% CO_2_ atmosphere.

### 4.4 Animals

The animal experiments in this study were approved by the Ethics Committee for Animal Care and Use of Kumamoto University and were performed in accordance with the guidelines. BALB/c mice were purchased from Japan SLC, Inc. (Hamamatsu, Japan) and kept under a 12‐h light/dark cycle.

### 4.5 Intracellular Uptake of GalNAc‐Modified Polymers

HepG2 cells (1.0 × 10^5^ cells/well in 24‐well plates) were seeded 24 h before treatment. The cells were washed twice with serum‐free medium, and GalNAc‐modified polymers (0.78–200 nm, 500 µL in DMEM) were added to each well and incubated at 37°C for 1.5–24 h. For a competitive inhibition study, free GalNAc (6.25–200 mm, 500 µL in DMEM) was added to each well, and the cells were washed twice with serum‐free medium and treated with 500 µL of DMEM containing free GalNAc (6.25–200 mm) and GalNAc‐modified polymers (25 nm) at 37°C for 1.5 h. For the study of HeLa cells, the relative expression levels of *ASGR1* mRNA in HepG2 and HeLa cells were measured via RT‐qPCR using the ReverTra Ace qPCR RT Kit (TOYOBO, Tokyo, Japan) and THUNDERBIRD SYBR qPCR mix (Takara Bio, Shiga, Japan) on a CFX/96/384/Connect (Bio‐Rad Laboratories, Inc., Hercules, USA) with 10 µm of the following primer sets: *Human ASGR1*, 5’‐GAAATTTGTCCAGCACCACATA‐3’ and 5’‐CAGTTCCTGGGGACAGAGC‐3’; and *human GAPDH*, 5’‐GCACCGTCAAGGCTGAGAAC‐3’ and 5’‐ATGGTGGTGAAGACGCCCAGT‐3’.

Following the treatment, the cells were washed twice with HBSS, peeled off using the trypsin‐EDTA method, collected by centrifugation, and dispersed in 10% FBS containing HBSS (500 µL). This washing procedure was repeated twice. The obtained cells were filtered through a nylon mesh and analyzed using a BD Accuri C6 flow cytometer (BD Biosciences Japan, Tokyo, Japan) and the BD Accuri C6 software (BD Biosciences Japan). Data from 1.0 × 10^4^ cells were analyzed.

### 4.6 LYTAC Activity

HepG2 cells (1.0 × 10^5^ cells/well in 24‐well plates) were seeded 24 h before treatment. The cells were washed twice with serum‐free medium, and 500 µL of DMEM containing a mixture of LYTACs (50 nm) and fluorescein isothiocyanate‐labeled BSA (FITC‐BSA, 250 nm) was added to each well and incubated at 37°C for 4 h. Subsequently, the cells were treated as described above and analyzed using a BD Accuri C6 flow cytometer and the BD Accuri C6 software. Data from 1.0 × 10^4^ cells were analyzed.

For in vivo evaluation, 50 µL of saline containing FITC‐BSA (133 µg) was intravenously injected into male BALB/c mice (4 weeks old). Subsequently, 50 µL of PBS‐containing LYTACs (10 times equimolar amounts of FITC‐BSA) was intravenously injected. Three‐hours after the LYTAC injection, the mice were sacrificed and perfused with HBSS (Ca^2+^ +) and 5% collagenase buffer (pH 7.5, Ca^2+^ +). The livers were collected, cut with scissors, and the cells were suspended using tweezers and pipetting. The suspended cells were filtered through a nylon mesh and centrifugated (4°C, 800 rpm, 5 min), and the obtained cell pellet was resuspended in 1% BSA solution and centrifugated (4°C, 800 rpm, 5 min). This washing procedure was repeated thrice to isolate the hepatocytes. The lysosomes were isolated using the Minute Lysosome Isolation Kit, and the isolated lysosomes were homogenized with the Minute Non‐Denatured Protein Solubilization Reagent (100 µL). The fluorescence of the homogenized solution was measured using a Synergy H1 microplate reader.

### 4.7 Intracellular Uptake and Stability of Cas9 RNP Complexes

HepG2 cells (1.0 × 10^5^ cells/well in 24‐well plates) were seeded 24 h before treatment. The cells were washed twice with serum‐free medium, and 500 µL of DMEM containing the ATTO550‐labeled Cas9 RNP complexes were added to each well and incubated at 37°C for 4 h. For the stability study, 10% containing medium was used. Subsequently, the cells were treated as described above and analyzed using a BD Accuri C6 flow cytometer and the BD Accuri C6 software. Data from 1.0 × 10^4^ cells were analyzed.

### 4.8 Biodistribution of Cas9 RNP Complexes

ATTO‐Cas9 RNP complexes (100 pmol) were injected intravenously into male BALB/c mice (4 weeks old). Two hours after administration, the mice were sacrificed and perfused with PBS and 4% paraformaldehyde. The organs (heart, lungs, liver, spleen, and kidneys) were collected and observed using an IVIS imaging system (PerkinElmer, U.S. LLC., Shelton, USA).

### 4.9 Genome Editing In Vivo

Cas9 RNP complexes (415 pmol) were injected intravenously into male BALB/c mice (4 weeks old). Seven days after the administration, the mice were sacrificed and perfused with PBS. The liver was collected and homogenized at 24 000 rpm using TRIzol reagent. The total RNA was isolated from the liver in accordance with the manufacturer's protocol. The expression levels of *TTR* mRNA were measured via RT‐qPCR using the ReverTra Ace qPCR RT Kit and THUNDERBIRD SYBR qPCR mix on a CFX/96/384/Connect with 10 µm of the following primer sets: *Murine TTR*, 5’‐ CATGAATTCGCGGATGTG‐3’ and 5’‐GATGGTGTAGTGGCGATGG‐3’; and *Murine GAPDH*, 5’‐TGACCTCAACTACATGGTCTACA‐3’ and 5’‐CTTCCCATTCTCGGCCTTG‐3’.

### 4.10 Data Analysis

Data were presented as mean ± standard error. Statistical significance was assessed using the Student's t‐test for two‐group comparisons and Scheffe's test following an analysis of variance for multiple comparisons.

## Author Contributions

**Toru Taharabaru**: Conceptualization, data curation, formal analysis, funding acquisition, investigation, methodology, validation, visualization, writing – original draft, and writing – review and editing. **Keiichi Motoyama**: Funding acquisition, methodology, resources, and supervision. **Yuting Wen**: Methodology, supervision, and writing – review and editing. **Zhongxing Zhang**: Methodology. **Xuehao Tian**: Investigation. **Jun Li**: Funding acquisition, methodology, resources, supervision, and writing – review and editing. **Taishi Higashi**: Conceptualization, funding acquisition, investigation, methodology, supervision, resources, and writing – review and editing. All the authors approved the final version of the manuscript.

## Funding

This study was partially supported by Platform for All Regions of Kyushu & Okinawa for Startup‐ecosystem (PARKS), a Grant‐in‐Aid for Scientific Research (C) from the Japan Society for the Promotion of Science (JSPS, grant number 21K06672), a Grant‐in‐Aid for Early‐Career Scientists from JSPS (grant number 26K18525), a Grant‐in‐Aid for JSPS Research Fellows (grant number 21J20999), the JSPS Overseas Challenge Program for Young Researchers, the New Pharmaceutical Technology and Engineering Foundation, and an Academic Research Fund Tier 2 Grant from Singapore's Ministry of Education (grant No. T2EP30122‐0040).

## Conflicts of Interest

The authors declare no conflicts of interest.

## Supporting information




**Supporting File**: advs75996‐sup‐0001‐SuppMat.docx.

## Data Availability

The data that support the findings of this study are available from the corresponding author upon reasonable request.
