# Supplementary material for: Molecular Mobility of N‐Acetylgalactosamine‐Modified Cyclodextrins on a Polyrotaxane for Highly Efficient Liver Targeting of Antibody Chimeras and Genome‐Editing Ribonucleoproteins
Source: Adv Sci (Weinh). 2026 Jun 11;13(49):e75996. doi: 10.1002/advs.75996 (PMC13336834; doi:10.1002/advs.75996)
Supplement: Supplementary file 1 [file ADVS-13-e75996-s001.docx]

Supporting Information

**Molecular Mobility of N-Acetylgalactosamine-Modified Cyclodextrins on a Polyrotaxane for Highly Efficient Liver Targeting of Antibody Chimeras and Genome-Editing Ribonucleoproteins**

*Toru Taharabaru,^1,2^ Keiichi Motoyama,^1^ Yuting Wen,^2*^ Zhongxing Zhang,^2^ Xuehao Tian,^2^ Jun Li,^2*^ Taishi Higashi ^1*^*

^1^Graduate School of Pharmaceutical Sciences, Kumamoto-University, 5-1 Oe-honmachi, Chuo-ku, Kumamoto 862-0973, Japan.

^2^Department of Biomedical Engineering, National University of Singapore, 15 Kent Ridge Crescent, Singapore, 119276, Singapore

*Corresponding authors: Yuting Wen, Jun Li, and Taishi Higashi

[wenyuting@u.nus.edu](mailto:wenyuting@u.nus.edu) (Y.W.)

[jun-li@nus.edu.sg](mailto:jun-li@nus.edu.sg) (J.L.)

[higashit@kumamoto-u.ac.jp](mailto:higashit@kumamoto-u.ac.jp) (T.H.)

*Supplementary Figures and Tables*

Supplementary Figure 1. ^1^H-NMR spectrum of (Ac)GalNAc-spacer-NH_2_ in *dmso-d_6_* (400 MHz).

Supplementary Figure 2. ^13^C-NMR spectrum of (Ac)GalNAc-spacer-NH_2_ in *dmso-d_6_* (150 MHz).

Supplementary Figure 3. FAB-MS spectrum of (Ac)GalNAc-spacer-NH_2_.

Supplementary Figure 4. ^1^H-NMR spectrum of FAM-PRX in *dmso-d_6_* (400 MHz).

Supplementary Figure 5. ^13^C-NMR spectrum of FAM-PRX in *dmso-d_6_* (150 MHz).

Supplementary Figure 6. ^1^H-NMR spectrum of FAM-HP-PRX in D_2_O (400 MHz).

Supplementary Figure 7. 2D-NOESY ^1^H-NMR spectrum of FAM-HP-PRX in D_2_O (500 MHz, Mixing time = 300 msec). Highlighted circles show the cross-peak of PEG proton and anomeric proton of α-CD.

Supplementary Figure 8. ^13^C-NMR spectrum of FAM-HP-PRX in D_2_O (150 MHz).

Supplementary Figure 9. ^1^H-NMR spectrum of *mono*GalNAc-PRX in D_2_O (400 MHz).

Supplementary Figure 10. 2D-NOESY ^1^H-NMR spectrum of *mono*GalNAc-PRX in D_2_O (500 MHz, Mixing time = 300 msec). Highlighted circles show the cross-peak of PEG proton and anomeric proton of α-CD.

Supplementary Figure 11. ^1^H-NMR spectrum of *tri*GalNAc-PRX in D_2_O (400 MHz).

Supplementary Figure 12. Preparation of FAM-labeled DEX backbone.

Supplementary Figure 13. ^1^H-NMR spectrum of FAM-HP-DEX in D_2_O (400 MHz).

Supplementary Figure 14. ^1^H-NMR spectrum of *mono*GalNAc-DEX in D_2_O (400 MHz).

Supplementary Figure 15. ^1^H-NMR spectrum of *tri*GalNAc-DEX in D_2_O (400 MHz).

Supplementary Figure 16. Synthesis summary of (a) conventional *tri*GalNAc and (b) *mono*GalNAc-PRX in this study*.*

Supplementary Figure 17. ^1^H-NMR spectrum of HOOC-Ad-PRX in *dmso-d_6_* (600 MHz).

Supplementary Figure 18. ^1^H-NMR spectrum of HOOC-Ad-HP-PRX in D_2_O (500 MHz).

Supplementary Figure 19. ^1^H-NMR spectrum of DBCO-HP-PRX in D_2_O (500 MHz).

Supplementary Figure 20. ^1^H-NMR spectrum of DBCO-GalNAc-PRX in D_2_O (500 MHz).

Supplementary Figure 21. 2D-NOESY ^1^H-NMR spectrum of DBCO-GalNAc-PRX in D_2_O (500 MHz, Mixing time = 300 msec). Highlighted circles show the cross-peak of PEG proton and anomeric proton of α-CD.

Supplementary Figure 22. Preparation scheme of Fc-specific azide functionalized antibody.

Supplementary Figure 23. Chemical structures of DBCO-*tri*GalNAc.

Supplementary Figure 24. Size distribution of N_3_-antibody, DBCO-GalNAc-PRX, or GalNAc-PRX-LYTAC measured by using a Zetasizer Pro apparatus. This figure shows the representative data for 3 experiments.

Supplementary Figure 25. Intracellular uptake of FITC-BSA with GalNAc-PRX-LYTAC with or without free GalNAc, or GalNAc-unmodified PRX (DBCO-HP-PRX)-LYTAC. [FITC-BSA] = 100 nM. [Antibody] = 10 nM. [Free GalNAc] = 100 μM. n = 3. The mean fluorescence intensity of GalNAc-PRX-LYTAC + FITC-BSA was set at 100%.

Supplementary Figure 26. Intracellular uptake of DBCO-GalNAc-PRX or DBCO-*tri*GalNAc conjugated with N_3_-TEG-FAM. [DBCO-GalNAc-PRX] = 10 nM. [DBCO-*tri*GalNAc] = 10 nM. [N_3_-TEG-FAM] = 10 nM. n = 3. The mean fluorescence intensity of N_3_-TEG-FAM alone was set at 100%.

Supplementary Figure 27. Preparation scheme of Ad-cap-GalNAc-PRX.

Supplementary Figure 28. ^1^H-NMR spectrum of Ad-cap-GalNAc-PRX in D_2_O (500 MHz).

Supplementary Figure 29. 2D-NOESY ^1^H-NMR spectrum of Ad-cap-GalNAc-PRX in D_2_O (500 MHz, Mixing time = 300 msec). Highlighted circles show the cross-peak of PEG proton and anomeric proton of α-CD.

Supplementary Figure 30. Preparation scheme of βCD-cap-amino-PRX (5G).

Supplementary Figure 31. ^1^H-NMR spectrum of βCD-cap-PRX in *dmso-d6* (600 MHz).

Supplementary Figure 32. ^1^H-NMR spectrum of βCD-cap-amino-PRX (5G) in D_2_O (600 MHz).

Supplementary Figure 33. Thin layer chromatography of GalNAc-PRXs prepared in this study. A mixture of n-butanol: ethanol: water (5:4:3, v/v/v) was used as the eluent with anisaldehyde as the indicator.

Supplementary Table 1. Adjustment of modification ratio of GalNAc per αCD.

| Batch | CDI input  (/αCD mol) | GalNAc input  (/αCD mol) | Modified GalNAc//αCD |
| --- | --- | --- | --- |
| 1 | × 2.0 | × 6.0 | 0.12 |
| 2 | × 3.5 | × 10.5 | 0.24 |
| 3 | × 4.6 | × 13.8 | 0.36 |
| 4 | × 11.0 | × 33.0 | 0.90* |

* Same data shown in Table 1.

Supplementary Table 2. Characterization of DBCO-GalNAc-PRX.

| No. of αCD | Coverage (%) | (*mono*)GalNAc  /αCD | M.W.  (kDa) |
| --- | --- | --- | --- |
| 60 | 26.6 | ~1.0 | 120 |

Supplementary Table 3. Characterization of Ad-cap-GalNAc-PRX.

| No. of αCD | Coverage (%) | (*mono*)GalNAc  /αCD | M.W.  (kDa) |
| --- | --- | --- | --- |
| 86 | 21.5 | ~1.0 | 191 |

Supplementary Table 4. Characterization of βCD-cap-amino-PRX (5G).

| No. of αCD | Coverage (%) | Cystamine  /αCD | Diethylenetriamine  /αCD | M.W.  (kDa) |
| --- | --- | --- | --- | --- |
| 96 | 24.0 | ~0.5 | ~0.5 | 184 |

Supplementary Table 5. Supplier of chemicals or reagents used in this study.

| **Name** | **Supplier** | **Location** |
| --- | --- | --- |
| sgRNAs | Integrated DNA Technologies K.K. | Tokyo, Japan |
| Galactosamine pentaacetate | Combi-Blocks, Inc.- | San Diego, USA- |
| Trimethylsilyl trifluoromethanesulfonate | Sigma-Aldrich Co. LLC. | St. Louis, USA |
| 2-[2-(2-Chloroethoxy)ethoxy]ethanol | Tokyo Chemical Industry Co., Ltd. | Tokyo, Japan |
| Ethylenediamine (EDA) | Tokyo Chemical Industry Co., Ltd. | Tokyo, Japan |
| Polyethylene glycol (PEG) | Sigma-Aldrich Co., LLC. | St. Louis, USA |
| α-Cyclodextrin (αCD) | Nihon Shokuhin Kako Co., Ltd. | Tokyo, Japan |
| 5(6)-Carboxylfluorescein (FAM-COOH) | Sigma-Aldrich Co. LLC. | St. Louis, USA |
| Benzotriazol-1-yloxytris(dimethylamino)phosphonium hexafluorophosphate (BOP) reagent | Tokyo Chemical Industry Co., Ltd. | Tokyo, Japan |
| *N*-Ethyldiisopropylamine (EDIPA) | Tokyo Chemical Industry Co., Ltd. | Tokyo, Japan |
| Propylene oxide | Sigma-Aldrich Co., LLC. | St. Louis, USA |
| Dextran 70 (DEX) | Tokyo Chemical Industry Co., Ltd. | Tokyo, Japan |
| *N,N*-Carbonyldiimidazole (CDI) | Tokyo Chemical Industry Co., Ltd. | Tokyo, Japan |
| *N*-Hydroxysuccinimide (NHS) | Tokyo Chemical Industry Co., Ltd. | Tokyo, Japan |
| 1-(3-Dimethylaminopropyl)-3-ethylcarbodiimide hydrochloride (EDC) | Sigma-Aldrich Co., LLC. | St. Louis, USA |
| Tris[[2-(tert-butoxycarbonyl)ethoxy]methyl]metylamine | Combi-Blocks, Inc.- | San Diego, USA- |
| Trifluoroacetic acid | Tokyo Chemical Industry Co., Ltd. | Tokyo, Japan |
| 1,3-Adamantanediacetic acid | Sigma-Aldrich Co., LLC. | St. Louis, USA |
| Dibenzocyclooctyne (DBCO)-amine | Tokyo Chemical Industry Co., Ltd. | Tokyo, Japan |
| Anti-bovine serum albumin (BSA) antibody | Proteintech Group, Inc. | Rosemont, USA |
| Siteclick Antibody Azido Modification Kit | Thermo Fisher Scientific | Waltham, USA |
| *N*-[2-[2-[2-(2-Azidoethoxy)ethoxy]ethoxy]ethyl]-3',6'-dihydroxy-3-oxo-3H-spiro[isobenzofuran-1,9'-xanthene]-6-carboxamide (N3-TEG-FAM) | Tokyo Chemical Industry Co., Ltd. | Tokyo, Japan |
| 1-Adamantane acetic acid | Tokyo Chemical Industry Co., Ltd. | Tokyo, Japan |
| 6-O-α-(4-O-α-D-Glucuronyl)-D-glucosyl-β-CD (GUGβCD) | Ensuiko Sugar Refining Co.,Ltd. | Tokyo, Japan |
| Recombinant Cas9 protein | Takara Bio Inc. | Shiga, Japan |
| Opti-MEM | Thermo Fisher Scientific | Waltham, USA |
| Dulbecco’s Modified Eagle Medium (DMEM, high glucose) | FUJIFILM Wako Pure Chemical Corporation | Osaka, Japan |
| ReverTra Ace qPCR RT Kit | TOYOBO Co., Ltd. | Osaka, Japan |
| THUNDERBIRD SYBR qPCR mix | TOYOBO Co., Ltd. | Osaka, Japan |
| PCR primers | Hokkaido System Science Co.,Ltd. | Hokkaido, Japan |
| Minute Lysosome Isolation Kit | Invent Biotechnologies, Inc. | Plymouth, USA |
| Minute Non-Denatured Protein Solubilization Reagent | Invent Biotechnologies, Inc. | Plymouth, USA |
